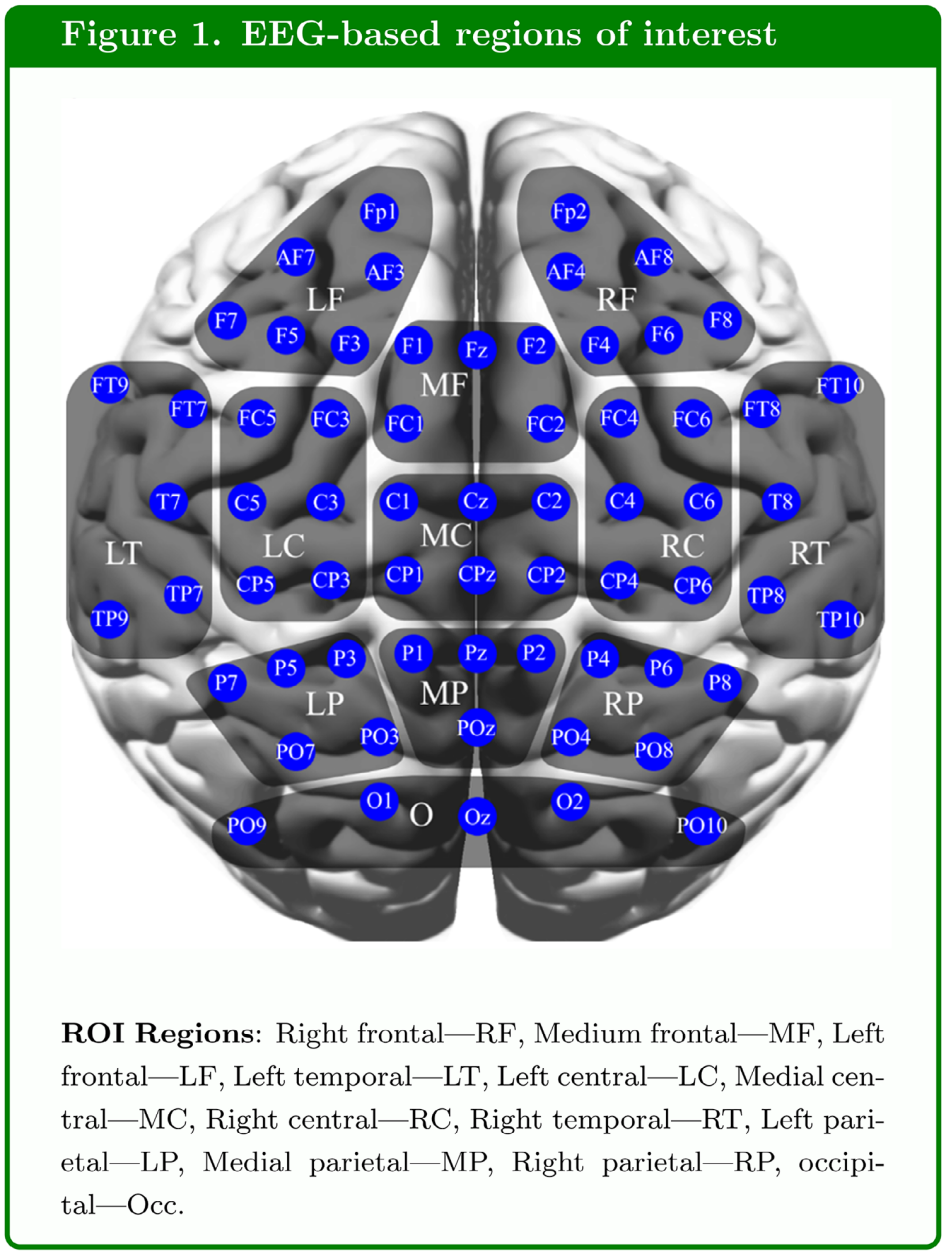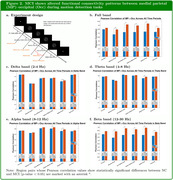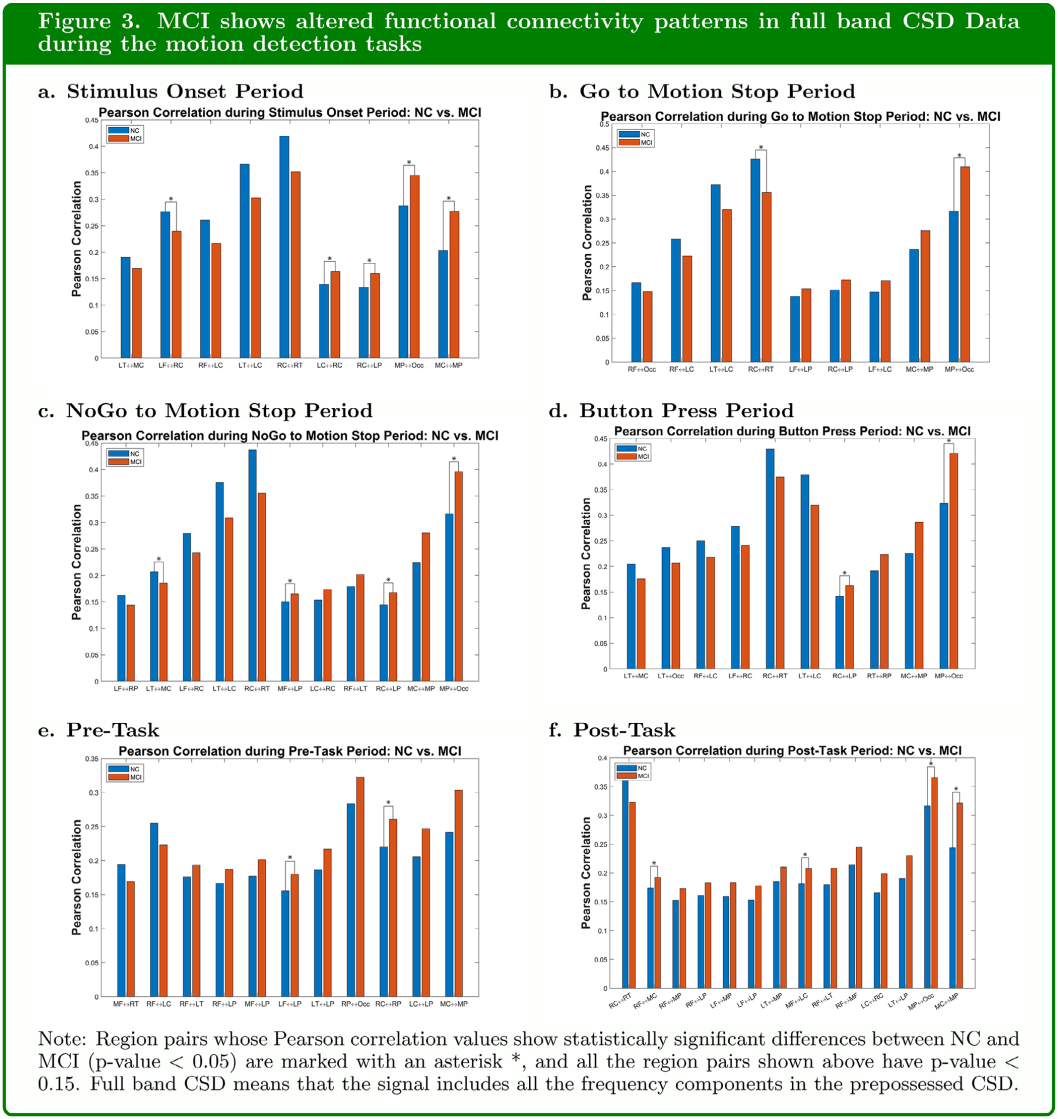# Altered functional connectivity patterns between medial parietal and occipital in mild cognitive impairment during motion detection tasks

**DOI:** 10.1002/alz.086032

**Published:** 2025-01-09

**Authors:** Ming Gu, Jinxian Deng, Boxin Sun, Voyko Kavcic, Tongtong Li, Bruno Giordani, Michigan Alzheimer's Disease Research Center

**Affiliations:** ^1^ Michigan State University, East Lansing, MI USA; ^2^ Wayne State University, Detroit, MI USA; ^3^ International Institute of Applied Gerontology, Ljubljana Slovenia; ^4^ Michigan Alzheimer's Disease Research Center, Ann Arbor, MI USA; ^5^ University of Michigan Medical School, Ann Arbor, MI USA

## Abstract

**Background:**

Patients with cognitive impairment are likely to suffer from weakening of functional connectivity between certain brain regions, which may often be accompanied by increased connectivity between some other regions, the latter of which may reflect the compensatory mechanisms of the brain. In this EEG‐based study, we investigate the differences in functional connectivity between persons with normal cognition (NC) and MCI patients in motion detection tasks.

**Method:**

Our research focuses on task‐based EEG (64‐channel) acquired at Wayne State University, where participants with subjective cognitive complaints were asked to perform a motion direction discrimination task. The current dataset includes 56 consensus‐diagnosed, community‐dwelling African Americans (ages 60‐90 years, 28 Normal Cognitions (NC) and 28 MCI patients) recruited through the Wayne State Institute of Gerontology and Michigan Alzheimer’s Disease Research Center.

We evaluated the functional connectivity at different time periods of the motion‐detection task across all the possible EEG region pairs using Pearson Correlation of the current source density (CSD). For each task trial, the successive time periods being examined included: (i) Pre‐Task resting‐state, (ii) Stimulus Onset to Go/NoGo indication, (iii) Go (or NoGo) indication to Motion‐Stop, (iv) Button‐Press period, (v) Post‐task resting‐state.

**Results:**

Our analysis indicates that when the full band CSD signals were considered, MCI showed statistically significant (p‐value < 0.05) and increased functional connectivity between MP↔Occ in all the time periods during the motion task. In comparison, the Beta band, which is believed to be related to attentional deficits in the visual performance among older persons, showed similar trends with the full band, and consistent and statistically significant increased functional connectivity between MP and PCC were observed in the Button‐Press period across all frequency bands.

**Conclusion:**

Our result suggests that MCI participants compensated for impaired connectivity in specific region pairs (see Figure 3) during the motion direction discrimination task by increasing the functional connectivity between MP and Occ to achieve comparable behavioral results to NC, and this may also be a particular indicator associated with MCI and AD pathology.

**Funding**: NSF‐2032709/Li; NIH‐1R21AG046637‐01A1/Kavcic and NIH‐1R01AG054484‐01A1/Kavcic; NIH‐P30AG072931/Paulson; NIH‐P30AG024824/Yung.